# Research on Dynamic Mechanical Properties of Silicon Carbide-Modified Concrete

**DOI:** 10.3390/ma18061374

**Published:** 2025-03-20

**Authors:** Tao Chen, Qingwei Chen, Yang Yu, Erlei Bai, Li Wang, Yanqin Guo, Ang Li

**Affiliations:** 1 Faculty of Engineering, Huanghe S&T University, Zhengzhou 450063, China; 15638862976@163.com (L.W.); 13673715828@163.com (Y.G.); 17303833890@163.com (A.L.); 2 Aviation Engineering School, Air Force Engineering University, Xi’an 710038, China; bwxkgy@163.com; 3 Mineral Resources Exploration Center of Henan Geological Bureau, Zhengzhou 450003, China; specter09@126.com

**Keywords:** silicon carbide, concrete, split Hopkinson pressure bar (SHPB), dynamic mechanics

## Abstract

This research investigates the dynamic mechanical properties of silicon carbide-modified concrete using a ∅ 100 mm large-diameter split Hopkinson pressure bar (SHPB). The effects of silicon carbide content, particle size, and strain rate on dynamic compressive strength, deformation, and energy dissipation characteristics were examined. The results indicate that both ordinary concrete and silicon carbide-modified concrete exhibit significant strain rate effects, with peak stress, impact toughness, and fracture degree progressively increasing as strain rate rises. The peak strain and ultimate strain of ordinary concrete decreased with increasing strain rate, while those of silicon carbide-modified concrete decreased initially before increasing again. At a strain rate of 180 s^−1^, the concrete fracture morphology showed almost no occurrence of cement paste connecting adjacent aggregates. The addition of silicon carbide directly increased the strength of aggregates at the micron scale, thereby enhancing the concrete’s load-bearing ability under high-velocity impact loads. Therefore, the modifying effect of coarse-fineness high-strength silicon carbide particles on the strength and deformation characteristics of concrete was more evident under high strain rate conditions.

## 1. Introduction

Concrete, as an inexpensive, easily sourced, and relatively strong building material, has been widely used in various military protective structures, and the study of its mechanical properties has gradually advanced [1]. In war environments, building materials are required to withstand large dynamic impact loads such as explosions and shocks [2]. This has driven the study of the mechanical response mechanisms and theories of concrete materials under high-speed explosive impact loading [3]. During this process, the concept of strain rate was introduced and gradually applied by many researchers in their studies [4].

Around the world, a systematic and common understanding has been established concerning the principles and modifications of SHPB technology [5], stress wave propagation and dispersion [6], the strain rate effect [7] on concrete, and stress wave effects [8]. The bar technique published in 1914 was developed to roughly determine the shapes of stress pulses produced by bullet impact and explosions [9]. The research techniques and theories regarding the dynamic mechanical properties of concrete materials were relatively mature [10]. Al-Salloum [11] investigated the dynamic behavior of concrete under high strain rates using SHPB testing, emphasizing the effects of specimen geometry and strain rate on concrete strength and failure modes. Cao Jixing [12] conducted in-depth studies on the numerical simulation and constitutive models of SHPB. Pham [13,14] incorporated rubber particles into concrete and conducted dynamic mechanical performance tests using a 100 mm diameter SHPB device. The results showed that the modified concrete exhibited improved impact resistance, and its strain rate sensitivity increased with the amount of rubber added. Tao [15] examined the effects of carbon fiber and carbon nanofiber on concrete using SHPB, revealing that carbon fiber enhanced ductility and energy dissipation, while carbon nanofiber improved strength and pore structure. Hu Shisheng [16] conducted dynamic mechanical performance tests on concrete using a 74 mm diameter SHPB device, and, in the same year, conducted a study on the dispersion effect of large-sized specimens [17]. The SHPB device requires one-dimensional assumptions for data processing, but for large-diameter pressure bars, two-dimensional effects, also known as dispersion effects or lateral Poisson’s effects, are likely to occur.

Silicon carbide-modified concrete is a new type of building material that incorporates silicon carbide into concrete to enhance its performance [18]. Due to the excellent properties of silicon carbide, such as high hardness, wear resistance, high-temperature resistance, good chemical stability, and excellent antioxidation properties [19], its incorporation as a modifier into traditional concrete can significantly enhance the overall performance of the concrete [20]. For example, silicon carbide concrete has properties such as wear resistance and high strength, making it widely researched for use in wear-resistant floors [21]. On the other hand, silicon carbide concrete also possesses certain wave-absorbing properties, and research on its electromagnetic shielding performance is extensive [22,23]. According to Idrees [24], silicon carbide was shown to significantly enhance the bending strength and durability of concrete composites, particularly in improving compressive strength and minimizing cracking. Kim [25] found that silicon carbide composite aggregates could significantly improve the thermal energy storage performance of concrete, especially after combining phase change materials with silicon carbide, which allowed for effective storage and release of thermal energy. Pundienė [26] found that silicon carbide aggregates notably improved the density, compressive strength, and fire resistance of refractory concrete, and effectively minimized deformation under high-temperature conditions. Research by Qiu [27,28] has shown that silicon carbide can influence the heat absorption properties and de-icing efficiency of concrete. Li Yongsheng’s study [29] demonstrated that silicon carbide concrete grounds had 3 to 5 times greater wear resistance compared with ordinary concrete floors. However, studies on the mechanical properties of silicon carbide concrete are still in the early stages. Wang Ruiyan [30] conducted mix design and static tests on silicon carbide concrete, and the results indicated that the addition of silicon carbide enhanced the workability of concrete and improved the uniformity of the spatial structure of hydration products, thereby enhancing the strength of the concrete. Experimental results from Xu Jinyu [31] also showed that silicon carbide can enhance the static compressive and flexural strength of concrete within a certain range.

It is clear that the research on silicon carbide in cement-based composites is in its initial phase [32], with the majority of studies concentrating on enhancing the electromagnetic shielding properties of concrete and conducting static mechanical tests. However, the research on the improvement patterns and mechanisms of silicon carbide in dynamic mechanical properties of concrete have not been sufficiently systematic and thorough. Moreover, as a particulate material, silicon carbide does not suffer from the dispersion issues, clumping during preparation, and uneven distribution that are common with fiber-based materials, making it more likely to achieve better improvements in certain properties. Based on this, this study used a comprehensive experimental approach to design tests that focused on the factors of silicon carbide content, fineness, and strain rate. Building on the theory of, and experimental research on, high strain rates in concrete, this study investigated the high-strain-rate dynamic mechanical properties of concrete modified with high-strength silicon carbide particles, focusing on the modification role of silicon carbide in this process and exploring its modification mechanism.

## 2. Experiment

### 2.1. Experimental Materials and Sample Preparation

The materials used in this study included cement, crushed stones, sand, water, superplasticizer, and silicon carbide. Ordinary Portland cement (P.O.) with a model number of 425R early strength cement was selected. The coarse aggregates were single-sized crushed stones with a maximum nominal diameter of 20 mm, and the smaller stones were limestone crushed stones. An 8:2 ratio of large and small stones was used. The fine aggregate was natural sand from the Bahe River, with a fineness modulus between 3 and 2.3. The test water came from the Baqiao District of Xi’an. The water reducer used was a polycarboxylate-based high-efficiency superplasticizer produced by Shanghai Chenqi Chemical Co., Ltd., Shanghai, China, with a water reduction rate of 28%. Silicon carbide had a density of 3.2 g/cm^3^ and a purity greater than 99%.

**Figure 1:**
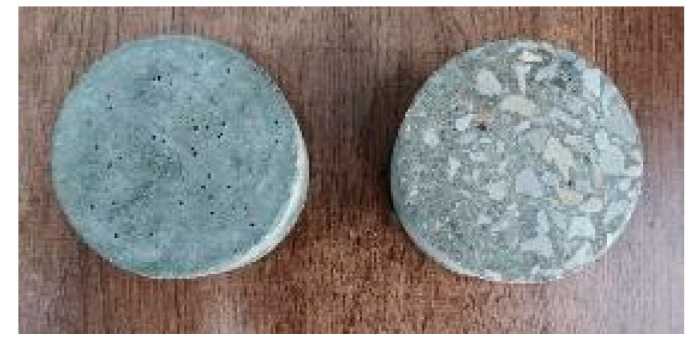
Comparison of specimens before and after polishing.

**Figure 2:**
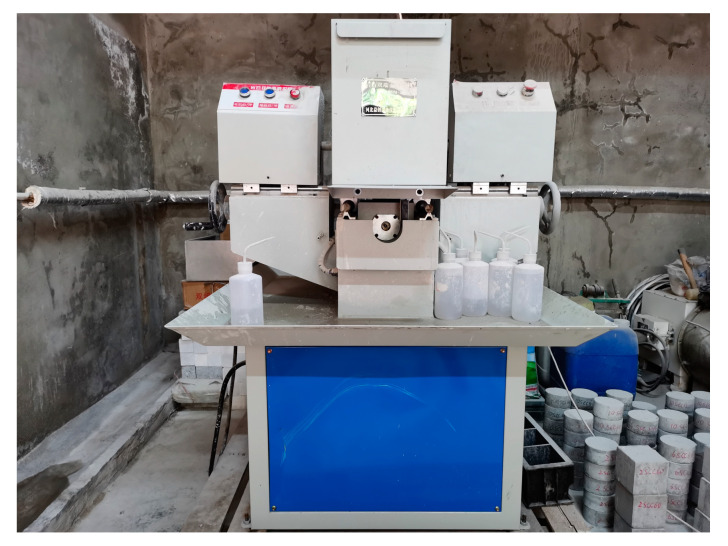
Double-end grinding machine.

Following the standard [33] that non-reactive admixtures in ordinary Portland cement should not exceed 10%, this study set the silicon carbide content to be 2%, 6%, 10%, and 14% by cement mass. Concrete samples were fabricated using the mix proportions shown in Table 1, with specimen dimensions of 100 mm in diameter and 50 mm in thickness, taking the form of cylinders. The preparation process strictly adhered to the following procedure: “cleaning the mold, oiling, pouring, vibrating, and curing”. Initially, the mold was cleaned and treated with L-HM anti-wear hydraulic oil to avoid adhesion. The materials were then weighed according to the calculated mix ratio, mixed uniformly, and poured into the mold. Compaction was performed to eliminate air pockets and voids. Lastly, the specimens were cured in a constant temperature and high-humidity chamber (20 °C, 95% humidity) until the designated curing age. The curing age for the samples was 28 days, ensuring the specimens achieved the required material properties prior to dynamic testing. Following curing, the specimens were polished in accordance with the specifications for the Hopkinson pressure bar impact test (Figure 1). The left side of the figure shows the specimen before polishing, and the right side shows the specimen after polishing. The dimensional tolerance for the diameter and length of the polished specimen was no more than 0.2 mm. To fully ensure the stability and reliability of the experiment, the polishing was performed using a double-end grinding machine, as shown in Figure 2.

### 2.2. Experimental Equipment and Methods

**Figure 3:**
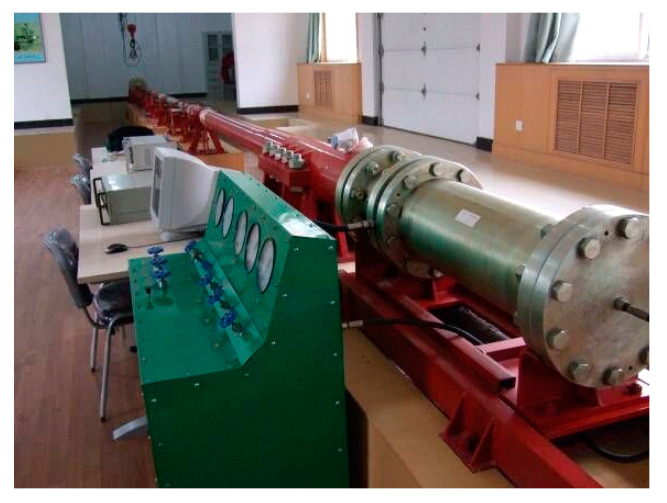
Split Hopkinson pressure bar uniaxial compression test system.

To investigate the influence of silicon carbide content and fineness on the modification effect of concrete under varying strain rates, silicon carbide content and fineness were chosen as key factors. Five levels of content (0%, 2%, 6%, 10%, and 14%) and five levels of fineness (0, 400 mesh, 600 mesh, 1200 mesh, and 2000 mesh) were used, with a curing age of 28 days for the modified concrete specimens. The experiments were performed using a 100 mm diameter SHPB (Figure 3). The impact velocity and strain rate of the specimens were controlled by adjusting the impact pressure of the striker bar [34]. Three pressure levels of 0.2 MPa, 0.3 MPa, and 0.4 MPa were applied, with the corresponding impact speeds and strain rates measured for each specimen group. This method is similar to the one described in reference [20]. Table 2 lists the naming conventions for the test specimens of silicon carbide-modified concrete.

## 3. Results and Analysis

### 3.1. Stress–Strain Curve

**Figure 4:**
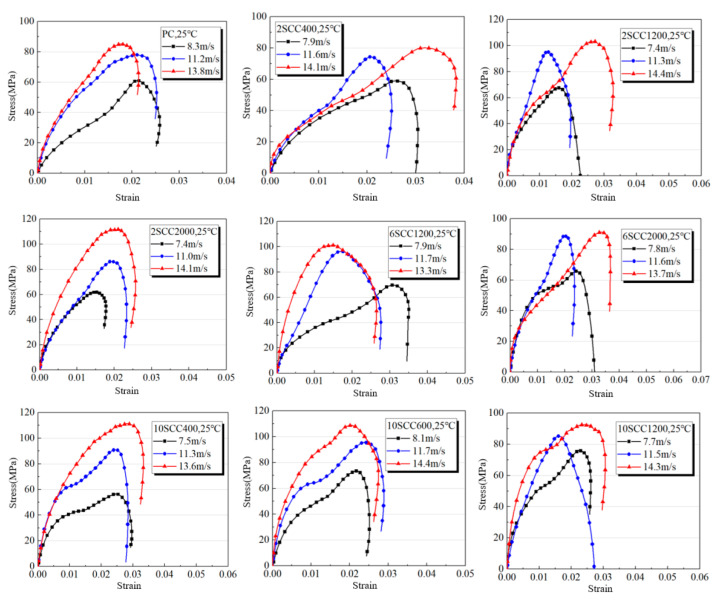

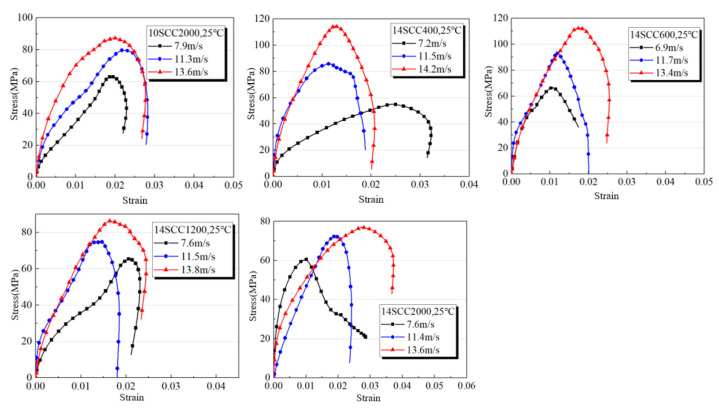
“Stress–strain” curve for various specimens’ dynamic compression.

Based on the experimental results, the stress–strain curves of silicon carbide-modified concrete under various loading rates, content levels, and fineness at similar strain rates are presented in Figure 4.

As shown in Figure 4, the stress–strain curves of the concrete specimens under uniaxial dynamic compression at high strain rates and under uniaxial static compression at low strain rates exhibited similar geometric shapes and trends. In the initial stage, the tangent slope of the three stress–strain curves was relatively high. As the strain increased, the tangent slope gradually decreased and stabilized at a relatively fixed value. Near the peak strain, the tangent slope rapidly decreased, and, after exceeding the peak strain, some residual strain remained with slight rebound. At high strain rates, the peak stress achieved by the concrete specimens was higher than at low strain rates, and the strain corresponding to the peak stress showed a similar trend. Additionally, it was observed that, within the scope of this experiment, the ultimate strain of ordinary concrete specimens decreased with the increase in strain rate.

Compared with the PC group specimens, the curves of all the silicon carbide-modified concrete specimens showed a larger gap between high and low impact speeds, indicating that the dynamic mechanical properties of concrete improved with the increase in impact velocity. As the strain rate increased, under the same silicon carbide content and fineness, the peak point of the stress–strain curve of silicon carbide-modified concrete shifted upward, indicating a significant strain rate strengthening effect on the peak stress of the concrete specimens.

### 3.2. Dynamic Strength Characteristics

**Figure 5:**
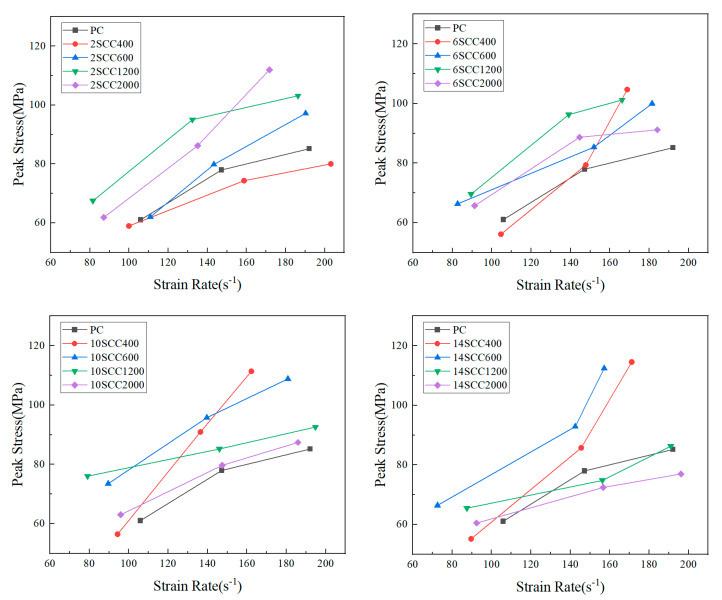
“Peak stress–strain rate” curves for various specimens of dynamic compression.

Dynamic compressive strength referred to the peak stress reached by concrete during impact compression, which was the maximum value in the dynamic stress–strain curve and could most directly reflect the load-bearing capacity of concrete. The higher the peak stress, the higher the failure limit of the material. Therefore, when evaluating the modification effect of silicon carbide on the dynamic mechanical properties of concrete, the peak stress needed to be analyzed. Figure 5 shows the effect of different silicon carbide fineness on the dynamic peak stress of concrete at various silicon carbide contents, reflecting the different modification effects of various silicon carbide fineness and contents on the concrete “peak stress–strain rate” relationship [35].

From Figure 5, it is evident that, under different silicon carbide contents, both ordinary concrete and silicon carbide-modified concrete exhibited an increase in dynamic peak stress with the rise in strain rate, demonstrating a noticeable positive correlation. This indicated that all concrete specimens displayed a significant sensitivity to strain rate. At the same strain rate, as the silicon carbide content increased, the compressive strength of coarse silicon carbide gradually increased, while the compressive strength of fine silicon carbide decreased. At 2% and 6% contents, the strain rate effect of coarse silicon carbide was lower than that of fine silicon carbide. However, at 10% and 14% contents, the strain rate effect of coarse silicon carbide was higher than that of fine silicon carbide. Therefore, at higher strain rates, fine silicon carbide yielded better strength modification results at lower contents, while coarse silicon carbide provided superior strength modification results at higher contents.

### 3.3. Deformation Characteristics Analysis

**Figure 6:**
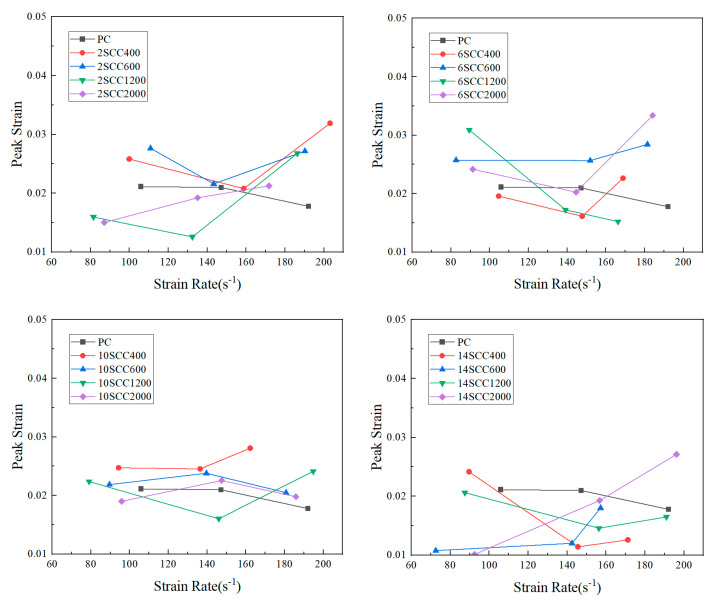
“Peak strain–strain rate” curves for various specimens of dynamic compression.

The deformation performance of concrete is typically defined as its capacity to endure deformation before a significant reduction in load-bearing capacity. Peak strains and ultimate strains under impact loading are analyzed to study dynamic compressive deformation. The peak strain is the strain corresponding to the peak stress on the stress–strain curve, indicating the maximum deformation the specimen could resist before failure. A larger peak strain suggests better deformation properties of the material [36]. As illustrated in Figure 6, the peak strains of the silicon carbide-modified concrete specimens were tested at various strain rates.

As shown in Figure 6, with the increase in strain rate, the peak stress of ordinary concrete specimens gradually increased while the peak strain gradually decreased. The addition of silicon carbide significantly improved this performance. At lower strain rates, the modification effect was not obvious, but. when the strain rate exceeded about 140 s^−1^, the peak strain of each group of silicon carbide-modified concrete specimens increased along with the peak stress. When the silicon carbide content was under 2% and 6%, and the fineness of silicon carbide was 600 mesh, its effect on enhancing the peak strain of concrete was better. When the silicon carbide content was under 10%, and the fineness of silicon carbide was 400 mesh, its enhancement effect on the peak strain of concrete was better. When the silicon carbide content was 14%, the addition of silicon carbide with a fineness of 2000 mesh was beneficial in increasing the peak strain of concrete at high strain rates. Overall, after the addition of silicon carbide, all groups of concrete specimens exhibited a strain rate threshold. Below this threshold, the peak strain decreased as the strain rate increased, while above this threshold, the peak strain increased with the strain rate. Ordinary concrete (PC) did not show such a threshold, and its peak strain consistently decreased with the increase in strain rate. Therefore, the incorporation of silicon carbide improved the deformation performance of concrete at high strain rates.

**Figure 7:**
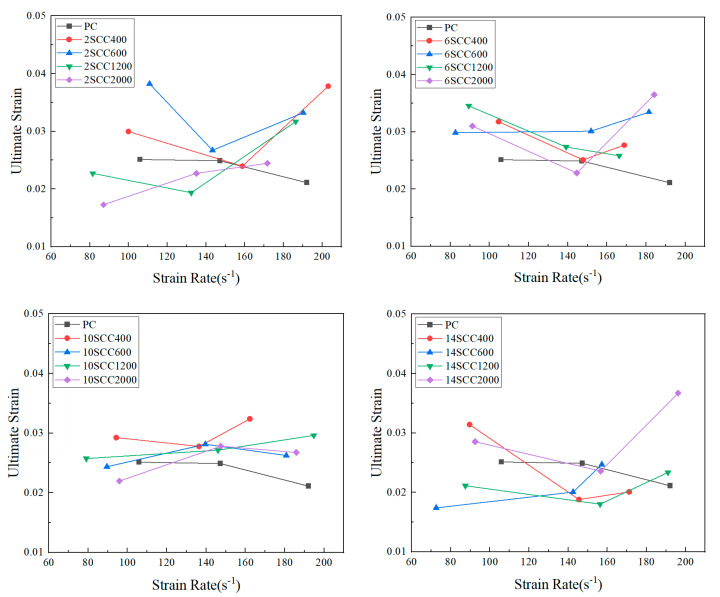
“Ultimate strain–strain rate” curves for various specimens of dynamic compression.

Ultimate strain refers to the maximum strain on the dynamic stress–strain curve, reflecting the ultimate deformation of concrete. Since concrete is a brittle material, its brittle characteristics become more pronounced at high strain rates, and it shows residual deformation even after the stress exceeds its ultimate strength. The larger the ultimate strain, the greater the residual deformation of the concrete, making ultimate strain an important indicator for evaluating the final deformation capacity and dynamic mechanical behavior of concrete [37]. Figure 7 shows the ultimate strain of silicon carbide-modified concrete specimens from each group at different strain rates.

Looking at the trend as a whole, with the increase in content, the ultimate strain values generally increased, indicating that the dosage within a certain range contributed to enhancing the material’s ultimate deformation capacity. As the dosage increased (from 2% to 14%), the ultimate strain of the specimens showed an overall upward trend, suggesting that the dosage contributed to improving the material’s deformation ability. During the increase in strain rate (from 80 s^−1^ to 200 s^−1^), the ultimate strain exhibited nonlinear changes. For lower dosages (such as 2% and 6%), the ultimate strain of some specimens increased initially with the strain rate but then decreased. For higher dosages (such as 10% and 14%), the ultimate strain of the specimens tended to increase monotonically with the strain rate, showing a stronger strain rate effect. Moreover, the trend of ultimate strain variation was similar to that of peak strain, albeit more complex. However, a strain rate threshold of 140 s^−1^ still existed, beyond which silicon carbide significantly improved both the peak strain and the ultimate strain of the concrete.

### 3.4. Energy Characteristics Analysis

The toughness of a material can be represented by the total energy it absorbs before reaching a certain load-bearing capacity. In this study, the total energy absorbed by concrete before the ultimate strain was considered as impact toughness. Impact toughness was the total energy absorbed by the concrete specimen before reaching the ultimate strain, expressed as the integral of the stress–strain curve, i.e., the area enclosed by the curve and the x-axis before the ultimate strain. This was influenced by both peak stress and ultimate strain, making it a comprehensive evaluation index with low data dispersion. The greater the impact toughness, the better the dynamic toughness of the concrete, the more energy it absorbed, and the stronger the modification effect of silicon carbide [38]. As shown in Figure 7, the impact toughness of each group of silicon carbide-modified concrete specimens at different strain rates is presented.

**Figure 8:**
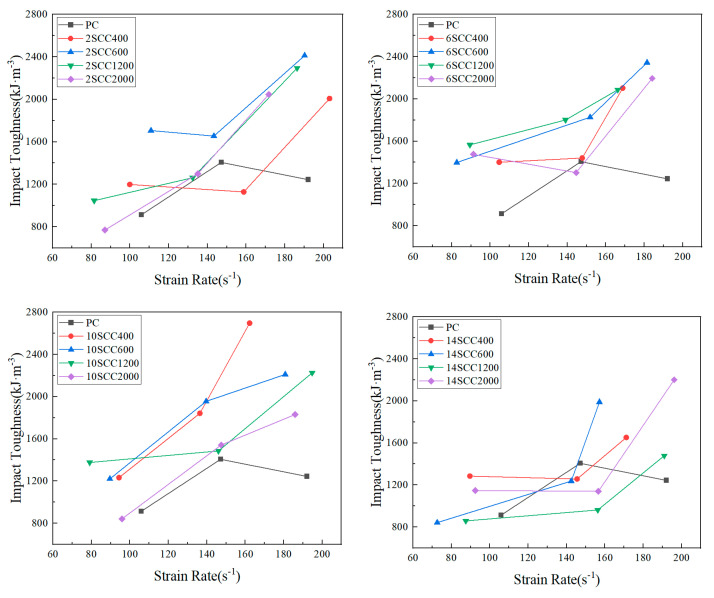
“Impact toughness–strain rate” curves for various specimens of dynamic compression.

From Figure 8, it can be seen that, after the incorporation of silicon carbide, the impact toughness of each group of specimens increased overall with the increase in strain rate. However, the impact toughness of PC increased first and then decreased with strain rate, indicating that the incorporation of silicon carbide significantly enhanced the specimens’ ability to absorb energy, and that the impact toughness of silicon carbide-modified concrete exhibited a significant strain rate effect. For concrete specimens modified with coarse-particle-size silicon carbide (400 mesh, 600 mesh), the impact toughness increased with the increase in silicon carbide content. However, after the content exceeded 10%, although the peak stress increased, the peak strain decreased, ultimately leading to a decrease in impact toughness. For concrete specimens modified with fine-particle-size silicon carbide (1200 mesh, 2000 mesh), the impact toughness remained relatively constant with the increase in silicon carbide content, but, at a content of 14%, it significantly decreased, even falling below the impact toughness of the PC group. Specifically, the 1200 mesh-modified concrete reached its optimal impact toughness at a content of 6%, while the impact toughness of the 2000 mesh-modified concrete gradually decreased as the content increased.

### 3.5. Optimal Content Analysis

From the analysis of the above experimental results, it was evident that the optimal choice of silicon carbide was influenced by three factors: content, fineness, and strain rate. There was no content and fineness that was optimal across all strain rate ranges, but there was a certain pattern in selecting the optimal fineness and content of silicon carbide.

To facilitate the analysis, this study discussed the optimal fineness and content of silicon carbide from the perspective of improving peak stress. Due to the difficulty in standardizing strain rates, the impact pressure of the impact rods was used as a reference for the control group, with the impact pressure of each rod representing a specific range of strain rates. Table 3 shows the optimal fineness corresponding to different contents across three strain rate ranges, while Table 4 presents the optimal contents corresponding to different fineness under three strain rates.

From Table 3, it can be seen that, as the strain rate increased, except for the specimens with a 2% dosage, the coarser the optimal fineness of silicon carbide, the more significant the improvement in the dynamic mechanical properties of concrete at high strain rates. This trend could be attributed to two main reasons. (1) Silicon carbide itself had high strength, which helped to improve the aggregate strength of concrete at high strain rates. The coarser the optimal fineness, the more pronounced the improvement. As the strain rate increased, the material’s demand for aggregate strength became higher, making the effect of coarse silicon carbide more significant. This was reflected in the fact that the optimal fineness of silicon carbide increased with the strain rate, and the higher the silicon carbide dosage, the more evident this phenomenon became. (2) At lower dosages, the strength of silicon carbide did not have a significant overall effect on the strength of the concrete. It mainly helped in filling pores and optimizing the mix design. In this case, the higher the strain rate, the better it was for silicon carbide to be finer. Therefore, at a 2% dosage, the optimal fineness of silicon carbide became finer as the strain rate increased.

From Table 4, it can be seen that, as the strain rate gradually increased, the optimal dosage of fine silicon carbide (1200 mesh, 2000 mesh) gradually decreased, while the optimal dosage of coarse silicon carbide (400 mesh, 600 mesh) gradually increased. The main reasons for this phenomenon were as follows: (1) Finer silicon carbide had a larger specific surface area and a more dispersed distribution. At high strain rates, it produced more microcracks with a higher probability, making defects caused by the larger number of cracks more noticeable as the strain rate increased; hence, the optimal dosage decreased. (2) Coarser silicon carbide had a more concentrated distribution and was more likely to generate higher aggregate strength. The higher the strain rate, the more important the role of aggregate strength became. Therefore, the advantage of coarse silicon carbide in improving aggregate strength became more evident, as the optimal dosage of coarse silicon carbide increased with the strain rate. From this perspective, it could be inferred that, in protective engineering facilities under high strain rate environments, higher dosages of coarse-particle-size silicon carbide should be used.

In summary, under low strain rate environments, finer silicon carbide (2000 mesh, 1200 mesh) helped fill the micron-sized pores within the concrete, thereby improving the concrete’s strength. However, under the influence of high strain rates and other special conditions, the optimal fineness of the concrete increased with a rising strain rate. Coarser silicon carbide demonstrated a more significant enhancement in the dynamic mechanical performance of concrete. Therefore, for protective engineering facilities that need to withstand impact loads, coarser silicon carbide (400 mesh and 600 mesh) should be preferred.

### 3.6. Mechanism of Action Analysis

Silicon carbide modified the dynamic mechanical properties of concrete in several aspects, including dynamic strength, dynamic deformation, dynamic energy, and fracture morphology. This study explored the mechanisms of enhanced dynamic mechanical performance of concrete and the modification mechanism of dynamic mechanical performance of silicon carbide-modified concrete.

1. Confining Pressure Factor

**Figure 9:**
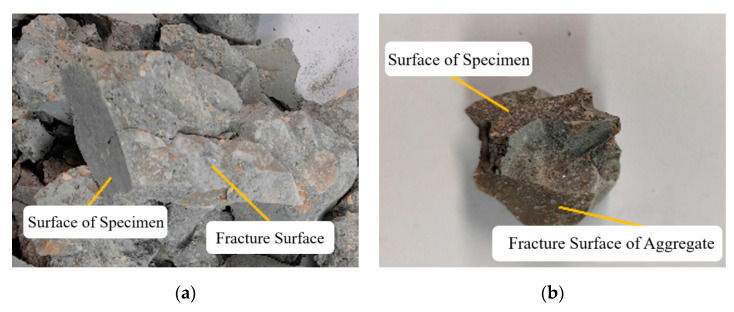
Analysis of confining pressure factors for each group of specimens. (**a**) 6SCC1200, 0.2 MPa. (**b**) 14SCC1200, 0.3 MPa.

In dynamic mechanical tests, the strain rate was relatively high, and the internal structures of the specimens had to complete their stress–strain responses in a short period, often resulting in mutual compression. As shown in Figure 9a, cracks expanded from the inside out, and the confinement pressure helped delay crack development during this process. Figure 9b shows that the aggregate failure surface was perpendicular to the specimen surface, indicating that the specimen was subjected to forces perpendicular to the axis during failure. Therefore, the central part of the concrete was actually subjected to confinement pressure from the edges, which increased the failure strength of the specimen.

2. Aggregate Factor

**Figure 10:**
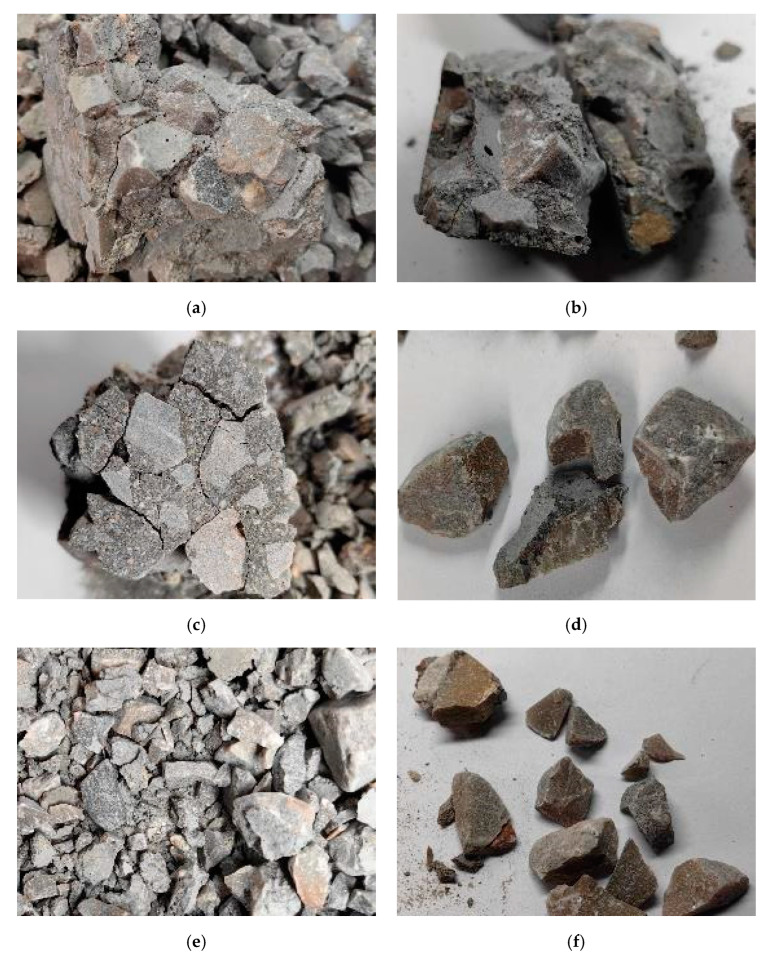
Analysis of aggregate factors for each group of specimens. (**a**) 2SCC400, 0.2 MPa. (**b**) 10SCC2000, 0.2 MPa. (**c**) 6SCC400, 0.3 MPa. (**d**) 14SCC1200, 0.3 MPa. (**e**) 2SCC400, 0.4 MPa. (**f**) PC, 0.4 MPa.

Figure 10 illustrates the damage analysis of aggregates in specimens under different strain rates. It was observed that, at an impact pressure of 0.2 MPa, cracks did not exhibit significant propagation along the aggregates, with cement paste still adhering to aggregates and minimal aggregate damage observed. At 0.3 MPa, cracks propagated distinctly along the aggregates, leading to more widespread aggregate failure. At 0.4 MPa, aggregates almost completely separated from one another, accompanied by substantial aggregate damage. In static mechanical tests, cracks had sufficient time to select the path of least energy consumption, typically breaking at the cement paste or interface, with minimal aggregate damage. However, under dynamic loading, cracks propagated through the interface, cement paste, and aggregates, making aggregate strength more critical to improving the dynamic mechanical strength of concrete.

3. Crack Factor

**Figure 11:**
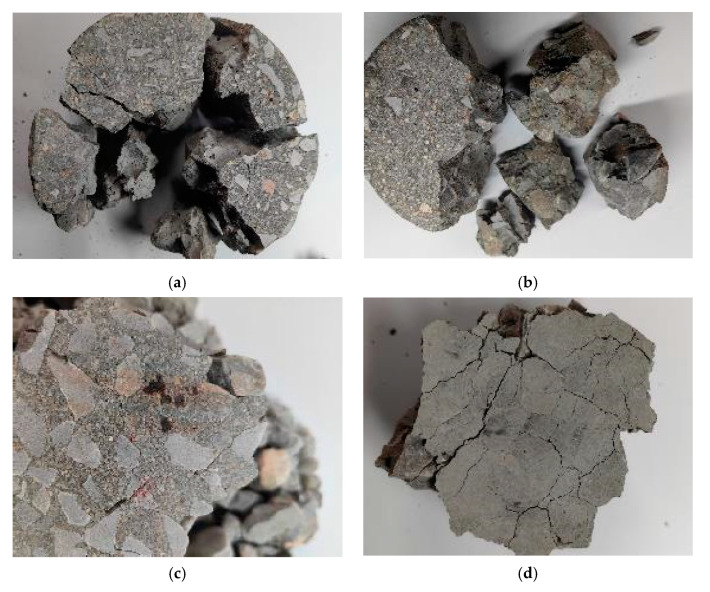
Main cracks of each group of specimens under different impact pressures. (**a**) 10SCC1200, 0.2 MPa. (**b**) 14SCC600, 0.2 MPa. (**b**) 14SCC600, 0.2 MPa. (**c**) 2SCC400, 0.3 MPa. (**d**) 10SCC1200, 0.3 MPa.

Figure 11 shows the analysis of the main crack development locations in some silicon carbide specimens under different impact pressures of the striking bar. It was observed that, under lower impact pressures, where strain rates were smaller, large concrete fragments were caused by a few through-cracks that extended outward from the center. As the strain rate increased, large fragments were mainly found in the core region of the concrete specimens, indicating that the damage had resulted from the simultaneous development of multiple micro-cracks. These micro-cracks were evenly distributed, making those closer to the outer edges more likely to have connected into continuous cracks. The reasons for this crack propagation phenomenon include the following: (1) At high strain rates, crack propagation speeds were significantly faster. In quasi-static tests, crack penetration took several seconds [39], but, in dynamic tests, it only took a few hundred microseconds. (2) High strain rates resulted in a significant increase in the number of propagating cracks. The fractured morphology showed that dynamic loading caused damage through the expansion of multiple micro-cracks, which required more energy and led to higher peak stress.

4. Cement Paste Factor

**Figure 12:**
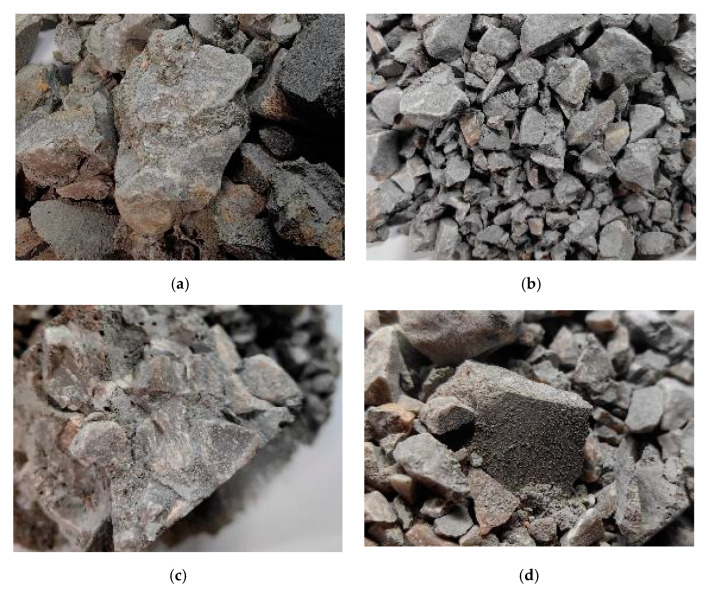
Analysis of cement paste factors for each group of specimens. (**a**) 2SCC400, 0.2 MPa. (**b**) 10SCC2000, 0.3 MPa. (**c**) 6SCC400, 0.3 MPa. (**d**) 2SCC400, 0.4 MPa.

Figure 12 shows the failure patterns of cement paste in specimens under different strain rates. At lower strain rates, some cement paste was able to retain its strength and maintained its role of bonding adjacent aggregates (Figure 12a). At higher strain rates, the cement paste was almost entirely fractured (Figure 12b). When the strain rate further increased, the cement paste was completely crushed into a powder-like state (Figure 12c). This indicated that, under impact loading, the failure of cement paste gradually transitioned from partial damage to complete destruction, evolving from crack-induced fractures to pulverization. Therefore, as the strain rate increased, the energy absorbed by the cement paste also increased, leading to an improvement in the energy absorption capacity of the concrete.

5. Pore Water Factor

Under impact loading at room temperature, the pore water within the concrete produced significant cohesive forces. Research indicated that concrete with higher humidity exhibited a more obvious strain rate effect, which was likely associated with the cohesive forces of the pore water. The presence of pore water restricted the relative movement of solid-phase materials across the pores, thereby improving the concrete’s dynamic strength and boosting its energy absorption ability under high-velocity impact loads.

## 4. Summary

This study used a comprehensive experimental method to design tests on three factors: the content of silicon carbide, its fineness, and its strain rate. The dynamic mechanical properties of concrete modified with high-strength silicon carbide particles at high strain rates were explored. The focus was on studying the modification effect of silicon carbide in this process and exploring its modification mechanism. The following conclusions were reached:

1. As the strain rate increased, both ordinary concrete and silicon carbide-modified concrete exhibited a significant strain rate effect, with gradually increasing peak stress, impact toughness, and degree of fragmentation.
2. From the perspective of strength characteristics, the optimal content and fineness of silicon carbide varied under different strain rates, but a notable pattern emerged. Within the scope of this study, the higher the strain rate, the greater the optimal content and the coarser the optimal fineness. Specifically, under high strain rates, coarse-grained and high-content silicon carbide exhibited the best modification effect. Overall, the optimal combination of content and fineness was 14SCC400, which, at an impact pressure of 0.4 MPa, achieved a strain rate of 171.17 s^−1^, and a dynamic peak stress of 114.51 MPa, representing a 34.43% improvement over PC concrete.
3. From the perspective of deformation characteristics, as the strain rate increased, the peak strain and ultimate strain of ordinary concrete gradually decreased, whereas those of silicon carbide-modified concrete first decreased and then increased after the strain rate exceeded 140 s^−1^. This indicated that the incorporation of large-particle high-strength silicon carbide effectively improved the deformation performance of concrete under high strain rates. From the perspective of fragmentation, when the strain rate reached 180 s^−1^, no cement paste connections between adjacent aggregates were observed, indicating that the cement paste interfaces within the concrete were almost entirely destroyed, and the specimen fragmented into cement paste powder and coarse aggregate pieces.
4. The relationship between the load-bearing capacity of concrete and the strength of aggregates became more pronounced under high strain rates. The incorporation of silicon carbide directly enhanced the aggregate strength at the micron level, thereby increasing the load-bearing capacity of concrete under high-speed impact loading. Coarse-grained high-strength silicon carbide particles exhibited more prominent modification effects on the strength and deformation characteristics of concrete under high strain rates.

## Figures and Tables

**Table 1:** Mix proportion design for concrete specimens (kg/m^3^).

Cement	Sand	Large Gravel	Small Gravel	Water	Superplasticizer
315.0	631.0	1053.6	263.4	137.0	0.63

**Table 2:** Naming for silicon carbide modified concrete specimens.

	400 Mesh	600 Mesh	1200 Mesh	2000 Mesh
0	PC			
2%	2SCC400	2SCC600	2SCC1200	2SCC2000
6%	6SCC400	6SCC600	6SCC1200	6SCC2000
10%	10SCC400	10SCC600	10SCC1200	10SCC2000
14%	14SCC400	14SCC600	14SCC1200	14SCC2000

**Table 3:** Optimal fineness at different contents.

Number	Impact Pressure	Strain Rate	Silicon Carbide Content			
			2%	6%	10%	14%
1	0.2 MPa	70–110 s^−1^	1200	1200	1200	600
2	0.3 MPa	130–150 s^−1^	1200	1200	600	600
3	0.4 MPa	160–200 s^−1^	2000	400	400	400

**Table 4:** Optimal amount at different fineness.

Number	Impact Pressure	Strain Rate	Silicon Carbide Fineness			
			400 Mesh	600 Mesh	1200 Mesh	2000 Mesh
1	0.2 MPa	70–110 s^−1^	2%	10%	10%	6%
2	0.3 MPa	130–150 s^−1^	10%	10%	6%	6%
3	0.4 MPa	160–200 s^−1^	14%	14%	2%	2%

## Data Availability

The original contributions presented in this study are included in this article, further inquiries can be directed to the corresponding authors.
